# Distribution Shifts of *Vaccinium* Berry Wild Relatives in China Under Climate and Land‐Use Changes

**DOI:** 10.1002/ece3.74154

**Published:** 2026-08-09

**Authors:** Ling Pang, Saijing Liu, Tongmei Yin, Lijuan Mu, Jiayi Song, Yin Liu, Siyi Li, Duo Ye, Mide Rao

**Affiliations:** ^1^ College of Life Sciences Zhejiang Normal University Jinhua China; ^2^ Naturalis Biodiversity Center Leiden the Netherlands; ^3^ Institute of Biology Leiden Leiden University Leiden the Netherlands

**Keywords:** berry wild relative species, climate and land‐use changes, dispersal limitation, long‐term conservation, persistent suitable areas, *Vaccinium*

## Abstract

Predicting species distributions under future environmental change is essential for understanding biodiversity responses and informing conservation planning. *Vaccinium*, a genus that includes important germplasm resources for many commercial berry crops, comprises diverse berry wild relatives (BWRs) in China, yet research on their responses to environmental changes remains limited. Here, we used occurrences of eight *Vaccinium* BWRs in China and ensemble species distribution models to predict their distributions in the 2070s under climate and land‐use changes (SSP126, SSP245, and SSP585) together with two dispersal scenarios (20 km/decade and full dispersal). We found stronger effects from climate‐related variables than land‐use variables on the distributions of the eight BWRs. The current potential distribution areas were largely consistent with known distributions, with generally high or moderate suitability. Most species would have their range size contracted, while two species were projected to have considerable range size increased. There was a trend of range shifts toward higher latitudes for most species. Based on projected persistent suitable areas, three northeastern distributed species would have their habitat limited in the Greater Khingan Mountains area, while five southern distributed species would harbor relative stability in their own specific areas. These findings enhance our understanding of how climate and land‐use changes may reshape the distributions of *Vaccinium* BWRs in China and provide valuable insights for their long‐term conservation and management.

## Graphical abstract

Using ensemble species distribution models, we projected the future distributions of eight Vaccinium berry wild relatives in China under climate and land‐use changes, as well as different dispersal scenarios. Most species were predicted to experience range contraction and higher latitude shifts, while climatic variables exerted stronger effects than land‐use variables on their distributions. Our results identify persistent suitable areas that may support the conservation and management of these berry wild relatives under future environmental changes.

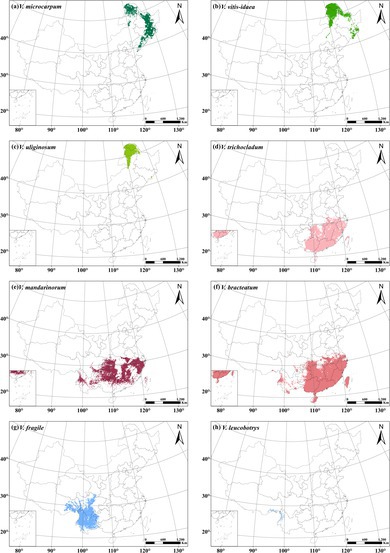

## 1 Introduction

Climate and land‐use changes are currently regarded as two major threats to global biodiversity (Peng et al. 2021). Numerous studies have shown that climate change has led to alterations in species' geographic distribution patterns, even causing extinctions of many species (Shi et al. 2023; Wiens and Saban 2025). Anthropogenic land‐use changes have modified the composition of communities, thereby resulting in habitat loss and biodiversity decline (Peters et al. 2019). According to the latest assessments from IUCN, the risk of global biodiversity loss remains at a high level (IUCN 2025). Therefore, investigating future changes in species' ranges under climate and land‐use scenarios, and developing corresponding conservation or management strategies, has become a major priority for preventing continued biodiversity decline. Crop wild relatives (CWRs) are plants evolutionarily related to cultivated crops and represent an important component of agrobiodiversity (Khoury et al. 2020). They have long been recognized as valuable genetic resources for crop improvement and food security, as they harbor traits that can be used in breeding new crop varieties (Kell et al. 2017). Beyond their agricultural importance, CWRs also contribute to plant diversity and may play important ecological roles in natural ecosystems. However, many CWRs are increasingly threatened in their native habitats, raising concerns not only about crop genetic erosion but also about the loss of biodiversity and ecosystem resilience (Akhalkatsi et al. 2010; Puchalka et al. 2023; Khoury 2024). Although previous studies have modeled the effects of climate and land‐use changes on species with economic or conservation importance (Song et al. 2021; Shen et al. 2021), the responses of CWRs to future environmental change remain comparatively understudied.

Species from *Vaccinium* have provided germplasm resources for many commercial crops (Migicovsky et al. 2022; Skrovankova et al. 2015), like blueberry, cranberry, and lingonberry. Beyond their edible and nutritional value, *Vaccinium* species also possess considerable economic importance in various non‐food sectors. Their bioactive compounds, particularly anthocyanins, flavonoids, and polyphenols, are widely applied in pharmaceutical, nutraceutical, and cosmetic industries due to their antioxidant, anti‐inflammatory, and anti‐aging properties (Van Hoed et al. 2009; Piazza et al. 2020; Martínez et al. 2024; Lorenzo et al. 2018). Apart from well‐known cultivated species, many other berry wild relatives (BWRs) from *Vaccinium* have considerable potential to be domesticated into new crops (Bao et al. 2024). Especially in China, a country that harbors 92 *Vaccinium* species, many of which hold great priority for commercial production, making it an important center for the conservation and study of these taxa (Narouei‐Khandan et al. 2017; Kopystecka et al. 2023). Researchers in China have made conscious efforts on *Vaccinium* BWRs' utilization, focused mainly on their pharmacological activity, chemical composition, cultivation methods, as well as their genetic relationships with commercial berries (Duan et al. 2022; Anderson et al. 2023). Nevertheless, how these BWRs will respond to future climate and land‐use changes remains poorly understood. Assessing the impact of future environmental changes on *Vaccinium* BWRs at the large spatial scale is in urgent need, which could be crucial for maintaining berry genetic diversity and better management for these species both within China and globally (Khoury et al. 2020).

Species Distribution Models (SDMs) have become a widely used tool for studying species distribution patterns (Guisan et al. 2013). By combining species occurrence data with corresponding environmental variables, SDMs can predict species' potential distributions across spatial and temporal scales (Pacifici et al. 2015; Liao et al. 2023). Moreover, based on the predicted patterns, identifying persistent suitable areas, that is, regions remaining suitable under both contemporary and future environmental scenarios (Beaumont et al. 2019), could be critical for conservation efforts. Serving as in situ climate refugia, these areas can help wild populations persist through extreme climate events (Baumgartner et al. 2018). In this study, based on ensemble SDMs, we aim to investigate the responses of the potential geographical distribution for eight *Vaccinium* BWRs in China to environmental changes. Specifically, our objectives include: (1) identify main environmental factors affecting their distribution patterns; (2) estimate the potential distributions of these species under current and future scenarios; (3) find persistent suitable habitats for them to provide reference for effective protection and sustainable management of berry resource.

## 2 Materials and Methods

### 2.1 Species Occurrence Data

**FIGURE 1:**
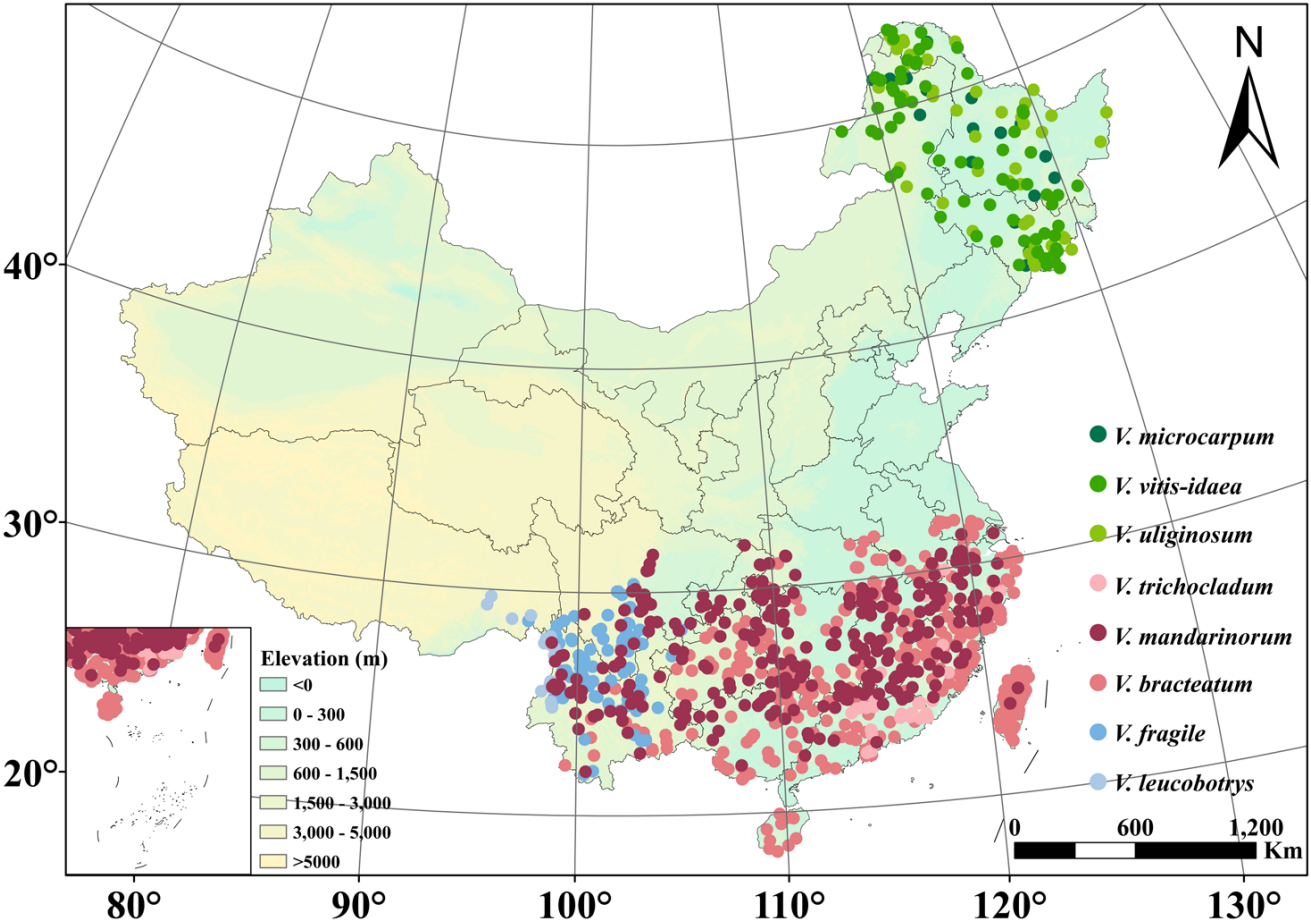
Distribution of the locations of eight *Vaccinium* berry wild relatives in China.

Eight species (i.e., *Vaccinium microcarpum* , *Vaccinium uliginosum* , *Vaccinium trichocladum*, *Vaccinium mandarinorum*, *Vaccinium leucobotrys*, *Vaccinium vitis‐idaea* , *Vaccinium fragile* and *Vaccinium bracteatum* ) in China were selected from the *Vaccinium* genus based on a comprehensive review of the *Flora of China*, local floras, and relevant scientific publications (Table S1). We then obtained their geographic occurrence data from Global Biodiversity Information Facility GBIF (https://www.gbif.org/), National Specimen Information Infrastructure NSII (https://www.nsii.org.cn/) and field investigations. Some specimen with detailed addresses were converted into latitude and longitude coordinates through Google Maps (http://www.gditu.net). To avoid overfitting, duplicate points were removed using the function “thin” in R package “spThin” within each grid with a spatial resolution of 5′ (Fick and Hijmans 2017; Duan et al. 2025). The final dataset contained 1213 spatial records for eight species (Figure 1; Table S2).

### 2.2 Climate Variables

Current (1970–2000) and future 2070s (average for 2061–2081) climate data were obtained from the WorldClim 2.1 (https://www.worldclim.org/) at a spatial resolution of 2.5′. Future climate data were based on the CMIP6 projections under three Shared Socioeconomic Pathways (SSP126, SSP245, and SSP585). To reduce multicollinearity, climate variables were divided into groups based on Pearson's correlation coefficients. Variables within each group were highly correlated (|*r*| > 0.8). Variables with high ecological relevance to the distribution of *Vaccinium* species were selected from each group, resulting in five variables: annual mean temperature (Bio1), max temperature of warmest month (Bio5), precipitation seasonality (Bio15), precipitation of warmest quarter (Bio18), and precipitation of coldest quarter (Bio19) (Hirabayashi et al. 2022; Puchalka et al. 2023) (Table S3).

### 2.3 Land‐Use and Elevation Variables

Land‐use types for both current and future (2070) scenarios were extracted from the FROM‐GLC database (Li et al. 2016). Snow‐covered areas and aquatic systems were excluded; we thus selected the remaining eight types (barren, cropland, forest, shrub, grass, impervious, urban greenspace, and wetland). Elevation data was also used in our models, due to many *Vaccinium* species were found in mountain areas (Wang 2016), and previous studies suggested elevation as a significant environmental factor affecting the distribution of plants (Luoto and Heikkinen 2007; Wang et al. 2025). Elevation data was obtained from WorldClim 2.1. Finally, five climate variables, eight land‐use variables, and elevation were used for SDMs analyses, all standardized at 10 km spatial resolution.

### 2.4 Species Distribution Models

Five species distribution models (generalized linear model, GLM; generalized boosting model, GBM; classification tree analysis, CTA; random forest, RF; and maximum entropy, MaxEnt) were employed to establish the relationship between species and environmental factors, as a single MaxEnt model may lack accuracy (Pan et al. 2025; Hao et al. 2019). To evaluate model performance, the distribution dataset was randomly divided into model training subsets (80%) and test subsets (20%) (Puchalka et al. 2023). After ten repetitions of this process for each species, we assessed the results based on true skill statistics (TSS) (Zhao et al. 2025). Results with TSS values > 0.5 were retained to project species distributions under current and future conditions (Thuiller et al. 2009) (Table S4). We then extracted the median predicted from all the models and further converted them into a binary layer representing presence‐absence distributions (Zhao et al. 2021). Since SDMs usually overpredict distributions, a 200 km buffered minimum convex polygon was set on the current species distribution range (Kremen et al. 2008). In addition, we assumed two dispersal scenarios: full dispersal and restricted 20 km/decade (Valladares et al. 2014; Liu et al. 2023). All these analyses above were performed using the R packages “biomod2” and “sf.”

### 2.5 Importance of Environmental Variables and Current Potentially Suitable Areas

The relative importance of 14 environmental variables was assessed to identify the key factors influencing the geographical distribution of each species, based on ensemble models generated through a randomization procedure (Kou et al. 2025). According to Jenks' natural breaks classification method (Zhang et al. 2025), the current potential suitable habitats for each species were classified into three classes, that is, low suitability, moderate suitability and high suitability.

### 2.6 Changes in Suitable Distribution

By comparing with the current potential distributions, future distribution areas were classified into stable, expansion, and contraction zones. To quantify the impact of environmental changes on the species distribution, we calculated the change in suitable area for each species: Range change rate = ((future suitable area—current suitable area) / current suitable area) × 100%. Centroid shift magnitude also serves as an important indicator for evaluating range changes (Fan et al. 2025). Therefore, we calculated centroid positions under both current and future scenarios to measure the distance and direction of centroid shift for each species. The analyses were implemented in the R package “geosphere.”

### 2.7 Persistent Suitable Areas Identification

Persistent suitable areas were defined as the overlap between the current potential suitable areas and the future suitable areas for each species, where the future suitable areas represented the intersection of suitable areas projected under SSP126, SSP245, and SSP585 for the 2070s (Ye et al. 2020). This analysis was carried out using ArcGIS 10.4.

## 3 Results

### 3.1 Current Potentially Suitable Areas

**FIGURE 2:**
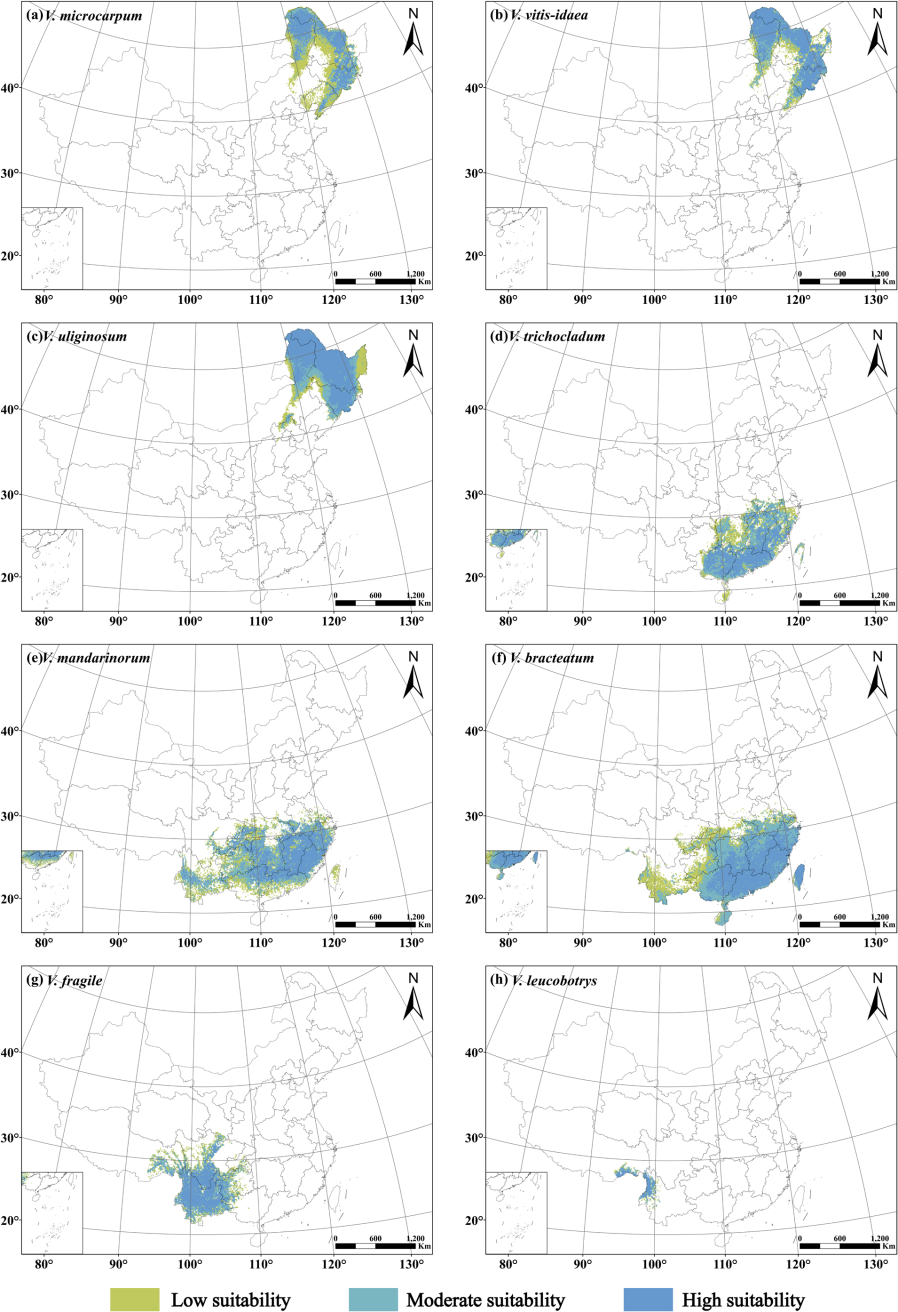
Current potentially suitable areas of *V. microcarpum* (a), *V. vitis‐idaea* (b), *V. uliginosum* (c), *V. trichocladum* (d), *V. mandarinorum* (e), *V. bracteatum* (f), *V. fragile* (g), and *V. leucobotrys* (h) in China.

The eight species showed various patterns of their current potentially suitable areas. The suitable areas for *V. microcarpum* (8.36 × 10^5^ km^2^), *V. vitis‐idaea* (8.13 × 10^5^ km^2^) and *V. uliginosum* (9.61 × 10^5^ km^2^) were found mainly in the northeastern part of China, with highly suitable areas concentrated in Heilongjiang, Jilin, and the northeastern part of Inner Mongolia (Figure 2a–c; Table S5). Suitable areas for *V. trichocladum* (9.68 × 10^5^ km^2^), *V. mandarinorum* (1.53 × 10^6^ km^2^) and *V. bracteatum* (1.74 × 10^6^ km^2^) occurred in regions south of the Yangtze River, with a high proportion of their optimal habitat located in Zhejiang, Jiangxi, Fujian, and parts of Guangdong and Guangxi (Figure 2d–f; Table S5). For *V. fragile*, its suitable habitats (7.81 × 10^5^ km^2^) were mainly concentrated in Yunnan and Sichuan (Figure 2g; Table S5). *V. leucobotrys*, with the smallest distribution area (9.67 × 10^4^ km^2^) among all species, was mainly located in Yunnan and Tibet (Figure 2h; Table S5).

### 3.2 Main Environmental Factors

**FIGURE 3:**
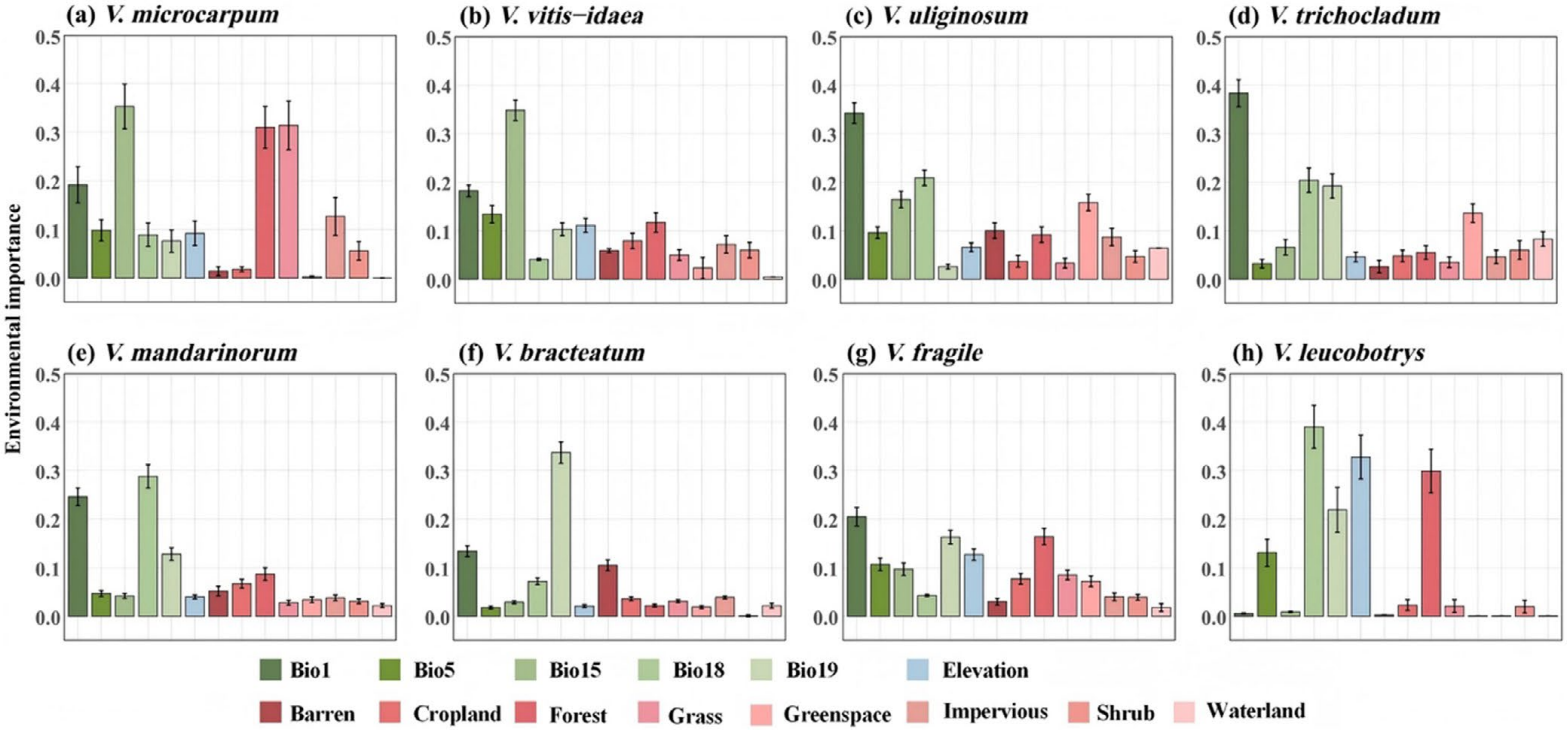
The relative importance of environmental variables for the distribution of *V. microcarpum* (a), *V. vitis‐idaea* (b), *V. uliginosum* (c), *V. trichocladum* (d), *V. mandarinorum* (e), *V. bracteatum* (f), *V. fragile* (g) and *V. leucobotrys* (h). Bio1: Annual mean temperature; Bio5: Max temperature of warmest month; Bio15: Precipitation seasonality; Bio18: Precipitation of warmest quarter; Bio19: Precipitation of coldest quarter.

Environmental factors with relative importance greater than 20% were regarded as key factors for distributions of *Vaccinium* BWRs. Climate variables were the primary determinants of most species' distributions. Precipitation seasonality played an important role in the distribution of *V. microcarpum* (Bio15, 35.3%) and *V. vitis‐idaea* (Bio15, 34.8%) (Figure 3a,b; Table S6). Annual mean temperature (Bio1) and precipitation of warmest quarter (Bio18) had the most pronounced influence on the distribution of *V. uliginosum* (Bio1, 34.2%; Bio18, 20.9%), *V. trichocladum* (Bio1, 38.3%; Bio18, 20.4%), and *V. mandarinorum* (Bio1, 24.6%; Bio18, 28.8%) (Figure 3c–e; Table S6). These two factors also limited the distribution of *V. fragile* (Bio1, 20.5%) and *V. leucobotrys* (Bio18, 38.9%) (Figure 3g,h; Table S6). In addition, precipitation of coldest quarter (Bio19, 21.9%) was vital to the distribution of *V. bracteatum* (Figure 3f; Table S6). Compared to climate variables, the effects of land‐use variables were relatively lower, except for *V. microcarpum* and *V. leucobotrys*, whose distributions were strongly affected by forest (31%; 29.9%) (Figure 3a,h; Table S6). Moreover, the distribution of *V. microcarpum* was also limited by the land‐use type of grass (31.4%) (Figure 2a; Table S6). Overall, although elevation had relatively low importance, it had a strong effect on the distribution of *V. leucobotrys* (32.8%) (Figure 3h; Table S6).

### 3.3 Distributional Range Changes in Suitable Areas

**FIGURE 4:**
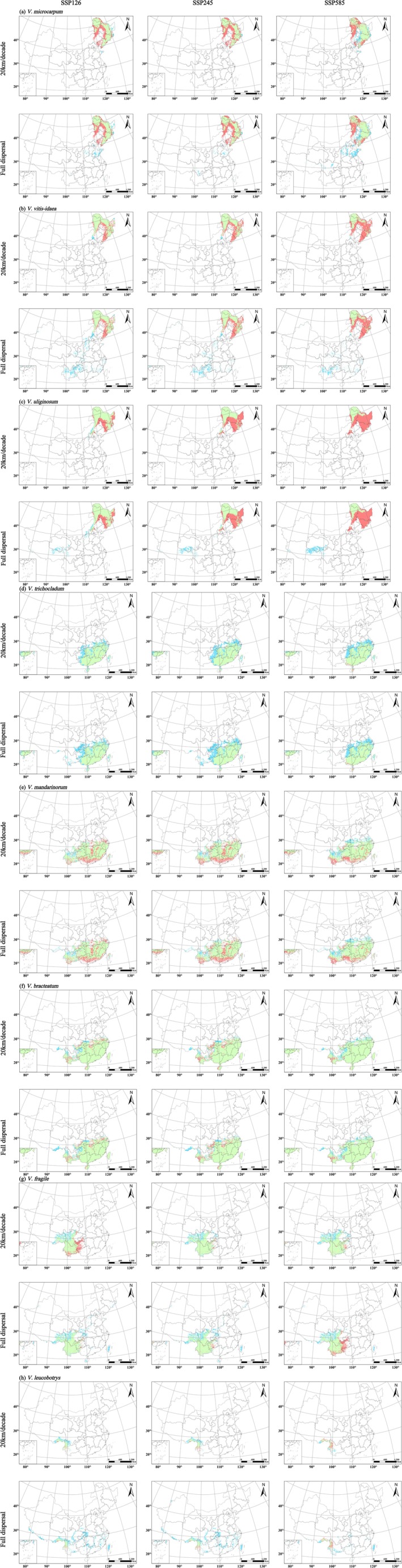
The changes in suitable areas of eight *Vaccinium* berry wild relatives in China between future and current potential distributions under three emission scenarios (SSP126, SSP245 and SSP585) and two dispersal scenarios (20 km/decade and full dispersal).

**FIGURE 5:**
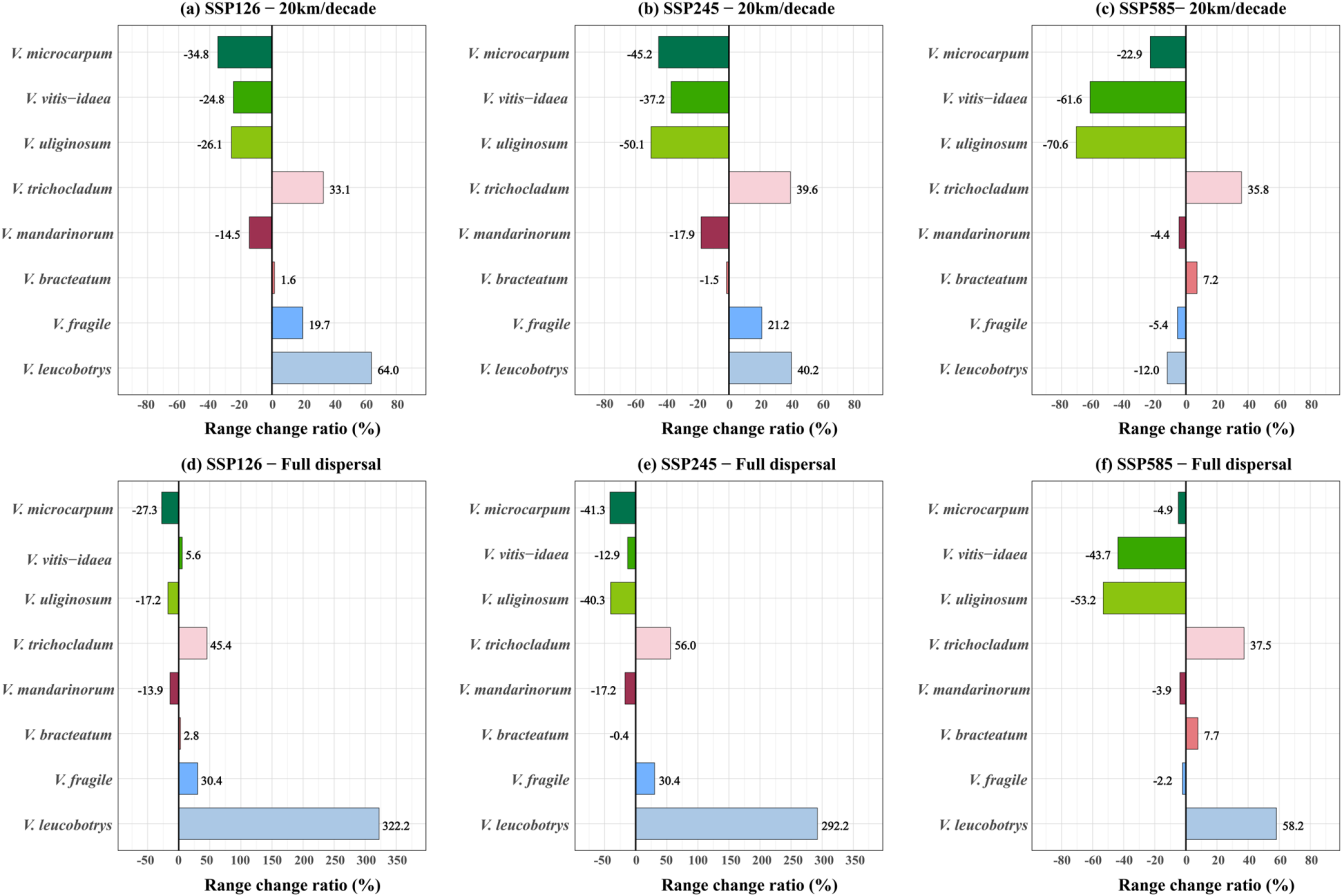
Ratio of range change in suitable areas of eight *Vaccinium* berry wild relatives under climate and land‐use change scenarios combining two dispersal scenarios.

All eight *Vaccinium* BWRs would undergo range shifts, while the degree of range expansion and contraction varied among species (Figures 4 and 5). Under the 20 km/decade dispersal scenario, both *V. microcarpum* , *V. vitis‐idaea* and *V. uliginosum* would experience habitat shrinking with limited expansion under all emission scenarios. Their habitat shrinking were projected mainly in Liaoning, Jilin and Heilongjiang (Figure 4a–c). Compared to other two species, *V. microcarpum* would face larger distribution area reduction in eastern part of Inner Mongolia (Figure 4a). The range loss reached the highest under the SSP245 scenario with 45.2% for *V. microcarpum* (Figure 5b; Table S7), while *V. vitis‐idaea* and *V. uliginosum* experienced the most severe range loss under the SSP585 scenario by 61.6%.and 70.6% (Figure 5c; Table S7). *V. trichocladum* would undergo considerable suitable area expansion under all emission scenarios (Figure 4d), with the largest increase of 39.6% occurring under the SSP245 scenario (Figure 5b; Table S7). With distribution contraction in the south and expansion in the north around its current suitable areas (Figure 4e), the potentially suitable areas of *V. mandarinorum* would decrease 14.5%, 17.9% and 4.4% under SSP126, SSP245 and SSP585 respectively (Figure 5a–c; Table S7). With almost equal range expansion and contraction, the overall suitable habitats for *V. bracteatum* remained relatively stable (Figure 4f). The expansion area for *V. fragile* concentrated on western Sichuan and eastern Tibet, while the contraction was mainly located at Guizhou (Figure 4g). Under SSP126 and SSP245, the distribution range for *V. fragile* would increase by 19.7% and 21.2%, while decrease at the ratio of 5.4% under SSP585 (Figure 5a–c; Table S7). *V. leucobotrys* was projected to contract mainly in Yunnan under all emissions scenarios. Range expansion occurred primarily in Tibet and northern Yunnan under SSP126 and SSP245, but in Tibet and southern Yunnan under SSP585 (Figure 4h). Its habitat range size was predicted to increase at a ratio of 64% and 40.2% under SSP126 and SSP245, while decrease by 12% under SSP585 (Figure 5a–c; Table S7). Under the full dispersal scenario, distribution range changes for most species exhibited similar trends to which under the 20 km/decade dispersal scenario, and generally showed lower degrees of contraction or higher range expansion ratio in suitable areas (Figure 5d–f; Table S7).

### 3.4 Geographical Centroid Shifts in Direction and Distance

**FIGURE 6:**
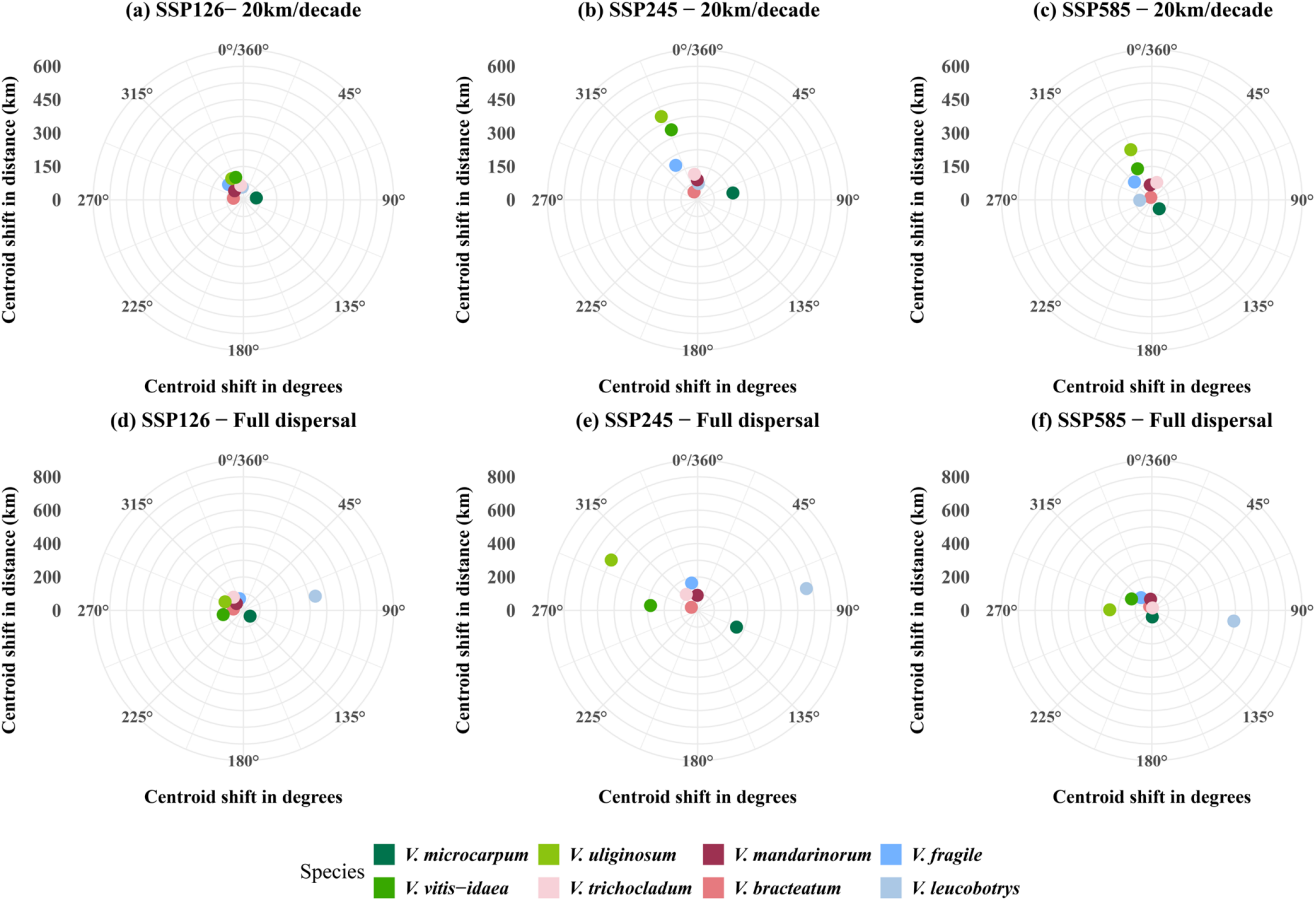
Centroid shifts in direction and distance of eight *Vaccinium* berry wild relatives between current and future distributions under climate and land‐use change scenarios combining two dispersal scenarios. The location of each dot indicates the shift of the centroid in direction and distance.

Centroids of distribution range for different species would shift differently in direction and distance (Figure 6; Table S8). Under the 20 km/decade dispersal scenario, the centroid of *V. microcarpum* was projected to shift eastward by 58 km and 162 km under SSP126 and SSP245, respectively, and southeastward by 52 km under SSP585 (Figure 6a–c; Table S8). A northward shift was observed for the centroid of *V. vitis‐idaea*, with a maximum shift distance of 335 km under SSP245 (Figure 6a–c; Table S8). *V. uliginosum* exhibited a similar direction shift by 109 km, 408 km and 244 km (Figure 6a–c; Table S8). The distribution center for *V. trichocladum* moved northward by 65 km, 115 km and 81 km under SSP126, SSP245 and SSP585 respectively (Figure 6a–c; Table S8). Compared to other species, the centroid of *V. mandarinorum* and *V. bracteatum* were relatively stable (Figure 6a–c; Table S8). The centroid of *V. fragile* was expected to shift toward northwest at the distance of 96 km, 183 km and 112 km, respectively (Figure 6a–c; Table S8). *V. leucobotrys* exhibited the westward shift of 73 km under SSP585 (Figure 6c; Table S8), and shifts under the other emission scenarios were comparatively limited (Figure 6a–c; Table S8). Under the full dispersal scenarios, most species showed similar centroid shifts, while the shift distance increased markedly for some species, especially *V. leucobotrys* (Figure 6d–f; Table S8).

### 3.5 Persistent Suitable Areas

**FIGURE 7:**
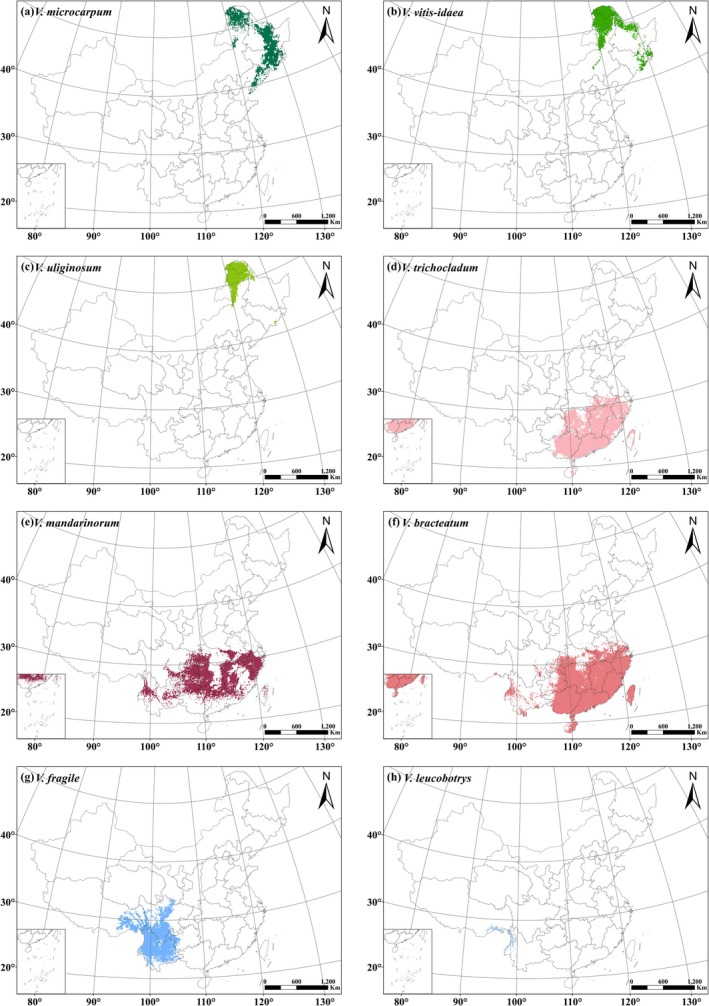
Persistent suitable areas of eight *Vaccinium* berry wild relatives under current and future scenarios.

The persistent suitable areas of eight *Vaccinium* BWRs located in different regions of China. The stable areas for *V. microcarpum* (3.35 × 10^5^ km^2^), *V. vitis‐idaea* (3.04 × 10^5^ km^2^) and *V. uliginosum* (1.97 × 10^5^ km^2^) were almost in the mountain areas in northeast China, including the Greater Khingan Mountains, Lesser Khingan Mountains, and Changbai Mountains (Figure 7a–c; Table S9). *V. trichocladum* (Figure 7d; Table S9) would have middle and lower reaches of the Yangtze River, the Guangdong—Guangxi region, and Fujian as its persistent suitable region (9.43 × 10^5^ km^2^). For *V. mandarinorum*, its persistent suitable areas (8.55 × 10^5^ km^2^) were concentrating in Zhejiang, Jiangxi, and the junction area of Hunan, Guizhou, and Chongqing, as well as Yunnan (Figure 7e; Table S9). *V. bracteatum* would hold its persistent suitable habitat (1.42 × 10^6^ km^2^) in most parts of South China, with the largest stable area among the eight species (Figure 7f; Table S9). For *V. fragile*, southwestern China, including Yunnan, Sichuan, and southern Tibet, provided its mainly stable area (5.86 × 10^5^ km^2^) (Figure 7g; Table S9). *V. leucobotrys* only harbored small stable areas (4.25 × 10^4^ km^2^) in the borders of Yunnan and Tibet (Figure 7h; Table S9).

## 4 Discussion

### 4.1 Current Potentially Suitable Areas and Main Environmental Variables

The current potential suitable areas for studied eight species were concentrated in northeastern China and the subtropical and tropical regions of southern China. This is generally consistent with the distribution pattern of *Vaccinium* in China, where species richness is relatively high in northeastern and southern China, while comparatively few species occur in the intervening regions (Fang et al. 1991). According to our results, precipitation variables played vital roles in shaping species' geographical distributions. Previous studies have shown that most *Vaccinium* species are particularly sensitive to drought events, because their shallow, root‐hairless roots restrict water and nutrient uptake from the soil (Ru et al. 2024). Thus, seasonal water deficits in the intervening regions may contribute to the observed distributional disjunction. In addition, the suitable areas of all eight species were located mainly within monsoon‐influenced regions, where summer rainfall coincides with rapid vegetative growth and fruit development (Selås 2000; Selås et al. 2015), thereby highlighting the importance of adequate water availability during these critical phenological stages (Lv et al. 2025).

However, the dominant precipitation variables differed between northeastern and southern distributed *Vaccinium* BWRs: precipitation seasonality was important for northeastern species, whereas southern species were associated primarily with precipitation during the warmest season or the coldest quarter. This divergence may reflect different biogeographic histories. *Vaccinium* is inferred to have originated in temperate North America and subsequently dispersed independently into north‐temperate and tropical Eurasia (Becker et al. 2024), potentially exposing colonizing lineages to different climatic filters. Such filtering may have promoted regional climatic specialization, increasing suitability within each group's respective precipitation regime while limiting establishment under the conditions occupied by the other, thereby reducing their distributional overlap (Kraft et al. 2015).

Annual average temperature was also a key factor for the distribution of most *Vaccinium* BWRs in this study, which may be due to many berry producing plants having thermoperiodic requirements during their growth period (Lv et al. 2025). Similarly, previous studies also found that temperature‐related variables are critical determinations for environmental fitness of *Vaccinium* species (Prevéy et al. 2020; Li et al. 2022). In the future, rising temperature may affect species distribution by shifting life history processes like germination and fruit ripening period (Hamilton et al. 2024).

Although land‐use variables were less important in shaping distributions for most studied species, forest habitat still had a remarkable influence on *V. microcarpum* and *V. leucobotrys*. This may be related to the unique ecological niche of the forests, where their rich biodiversity creates specialized microclimates, which are suitable for these species (De Frenne et al. 2019, 2021; Ashcroft and Gollan 2013). Moreover, like many other forest shrubs, the seed dispersal of *Vaccinium* BWRs is mainly mediated by biological mechanisms, especially the collection and storage of fruits by small forest animals (Du et al. 2009; Puchalka et al. 2023). Recent studies have reported that the decline in animal populations, driven by changes in forest area, could threaten the efficiency of animal‐mediated seed dispersal (Petrocelli et al. 2024). Therefore, the reduction of forest area due to global warming and anthropogenic activities may affect the seed dispersal of these species and lead to habitat shrinking. Previous studies have suggested elevation as an important variation influencing the distribution of *Vaccinium* species (Hamilton et al. 2024). In addition, our results showed that elevation does not have a strong impact on most *Vaccinium* BWRs, except for *V. leucobotrys*. The low contribution of elevation to the distribution of most species may indicate a relatively broad ecological tolerance across altitudinal gradients. These species occur across a relatively wide range of habitats and climatic conditions, suggesting that elevation per se does not strongly constrain their distributions. In contrast, the distribution of *V. leucobotrys* was strongly influenced by elevation, likely because of its narrow distribution range in the heterogeneous Hengduan Mountains and Himalayas (Zhu et al. 2025).

### 4.2 Future Potentially Suitable Areas Under Climate and Land‐Use Changes

Although most current habitats for our species were highly or moderately suitable, the majority of species in our study would be negatively affected by climatic and land‐use changes by the 2070s, with potential implications for the development of berry germplasm resources. We also found that southern distributed BWRs appeared more resilient to climate and land‐use changes than those distributed in the northeastern. Northeastern distributed BWRs would experience substantial range losses with limited expansion, especially under SSP585. Specifically, for *V. vitis‐idaea* and *V. uliginosum* , their suitable areas would be mainly retained in the Greater Khingan Mountains area, indicating conservation attention was urgently needed for them. Moreover, permafrost thaw induced by future warming was predicted to release inorganic nitrogen (Yun et al. 2023), which may alter climatic and soil conditions in Northeast China and provide an additional nitrogen source for *Vaccinium* BWRs. On the contrary, the suitable areas for southern distributed species would only experience obvious change along the margins of their current distribution ranges (Figure 4d–h), including both local habitat contraction and expansion. This finding suggests conservation and management strategies should mainly focus on the edges of these species' ranges. Furthermore, in contrast to the above negative impact from future environmental change, *V. trichocladum* was projected to expand its range with almost no contraction, indicating its wider tolerance to climate variability (Yu et al. 2019). This suggests that *V. trichocladum* holds substantial potential value for resource development. Consistent with previous studies (Auffret et al. 2010; Prevéy et al. 2020), the shift of these BWRs' geographical centroid would be mostly toward higher latitudes. This may be related to the general northward displacement of the climate zone caused by ongoing global warming (Zhao et al. 2023). Under the full dispersal scenario, however, *V. microcarpum* and *V. leucobotrys* exhibited a southward shift in their distributional centroids, primarily driven by the projected emergence of newly suitable habitats at lower latitudes, coupled with contractions in their currently suitable areas.

Our results showed that the setting of dispersal scenarios can reduce the overestimation by SDMs, thus improving the model accuracy (Liao et al. 2020). For example, under the full dispersal scenario, *V. vitis‐idaea* was predicted to expand into disjunct areas far from its current distribution, particularly in southern China. In contrast, under the 20 km/decade dispersal scenario, these southern disjunct areas were no longer predicted as suitable (Figure 4b). Similarly, for *V. leucobotrys*, the range expansion ratio was nearly five times and centroid shift distances were around nine times under the full dispersal scenario than that under the 20 km/decade (Figure 6d–f; Table S8). In fact, it is almost impossible for species to disperse to such disjunct areas without artificial assistance. Therefore, the predicted distributions under the dispersal scenarios of 20 km/decade were more reliable.

### 4.3 Persistent Suitable Areas for the Eight *Vaccinium* BWRs in China

Persistent suitable areas can help clarify the persistence of *Vaccinium* BWRs under future environmental change and highlight areas of conservation importance (Baumgartner et al. 2018; Beaumont et al. 2019). In our study, the three northeastern distributed species showed overlapping persistent suitable habitat in the Greater Khingan Mountains. These areas may function as future refugia and should therefore be considered long‐term conservation priorities for these northeastern taxa. Due to the extremely cold winters, few fruit cultivars can be grown in the Greater Khingan Mountains area (Li et al. 2022). In this context, our results provide valuable insights for the spatial planning of *Vaccinium* BWRs plantation in this area, and their cultivation promotion could contribute to both local economic development and the improvement of human well‐being. For most species distributed in southern China, current suitable areas were projected to remain relatively stable under future climate and land‐use changes, particularly for *V. trichocladum*, *V. bracteatum* , and *V. fragile*. This pattern suggests that these regions may continue to support the persistence of southern BWRs' populations under future environmental change. From an applied perspective, such taxa may also represent valuable genetic resources for improving the resilience of cultivated berry crops to climate change (Bao et al. 2024). By contrast, the persistent suitable area of *V. leucobotrys* was predicted to be extremely limited, highlighting its potential vulnerability and the importance of targeted in situ conservation under changing environmental conditions (Vincent et al. 2019). In addition, ex situ conservation technologies (i.e., storage, testing, and regeneration) are likely to be necessary to preserve this species in plant gene banks and botanical gardens over the long term (Khoury et al. 2019). Such efforts would help maintain genetic diversity and provide material for future restoration and reintroduction programs (Castañeda‐Álvarez et al. 2015).

## 5 Conclusions

In this study, we projected the combined effects of future climate and land‐use change on the distributions of eight *Vaccinium* BWRs in China. Our results showed that species distributions were driven primarily by climate variables, particularly annual mean temperature, precipitation of warmest quarter, and precipitation seasonality, although forest and grassland habitats also played important roles for some species. Under future scenarios, most species would experience a reduction in their distribution range, while some species were projected to undergo range expansion. Southern distributed species were generally less vulnerable to future climates than northeastern species, and the distribution centroids of most studied BWRs shifted toward higher latitudes. In addition, the majority of current potentially suitable areas for these BWRs would remain stable in the context of global warming, indicating their great potential for long‐term conservation and management. For the northeastern species, the Greater Khingan Mountains may represent a priority area for future conservation, as it constitutes a shared suitable habitat for all of them. Overall, these results provide new insights into the future distribution of *Vaccinium* BWRs in China and offer a scientific basis for conservation planning and resource management.

## Author Contributions

**Ling Pang:** conceptualization (equal), formal analysis (lead), investigation (lead), methodology (equal), visualization (lead), writing – original draft (lead). **Saijing Liu:** methodology (lead), writing – original draft (equal). **Tongmei Yin:** methodology (equal), writing – original draft (equal). **Lijuan Mu:** methodology (equal). **Jiayi Song:** investigation (equal), methodology (equal). **Yin Liu:** formal analysis (equal). **Siyi Li:** investigation (equal). **Duo Ye:** conceptualization (equal), writing – review and editing (equal). **Mide Rao:** conceptualization (equal), funding acquisition (lead), methodology (equal), supervision (lead), writing – original draft (equal), writing – review and editing (equal).

## Funding

This work was supported by the National Natural Science Foundation of China (32001224).

## Conflicts of Interest

The authors declare no conflicts of interest.

## Supporting information


**Table S1:** Species selection criteria and supporting references.
**Table S2:** Occurrence number for eight *Vaccinium* berry wild relatives.
**Table S3:** Pearson correlation coefficient matrix between selected climate variables.
**Table S4:** The performance of species distribution models for eight *Vaccinium* berry wild relatives. True skills statistics (the average of the five modeling approaches) are used in the current summary. Higher values indicate a higher predictive power of the models.
**Table S5:** The range size of the potential current suitable areas for eight *Vaccinium* berry wild relatives.
**Table S6:** Mean relative importance of each variable to the final SDMs of eight *Vaccinium* berry wild relatives. The values highlighted in bold are greater than 20% were selected as the main environmental factors for each species.
**Table S7:** The range change radio for eight *Vaccinium* berry wild relatives.
**Table S8:** Centroid shifts in direction and distance of eight *Vaccinium* berry wild relatives between current and projected distributions under climate and land use changes scenarios with two dispersal scenarios.
**Table S9:** Persistent suitable areas for eight *Vaccinium* berry wild relatives.

## Data Availability

All data used in this research has been uploaded to an open data repository via Figshare: https://figshare.com/s/82a7cbc123e591f4b8f0.
