# Correction to “Distribution Shifts of *Vaccinium* Berry Wild Relatives in China Under Climate and Land‐Use Changes”

**DOI:** 10.1002/ece3.74299

**Published:** 2026-09-03

Pang, L. , S. Liu , T. Yin , et al. 2026. “Distribution Shifts of *Vaccinium* Berry Wild Relatives in China Under Climate and Land‐Use Changes.” Ecology and Evolution 16, no. 8: e74154. 10.1002/ece3.74154.42572784 PMC13453431

In the published article, the color legend in Figure 4 indicating range contraction, range expansion, and stable range was missing. The corrected Figure 4 is shown below.

**Figure:**
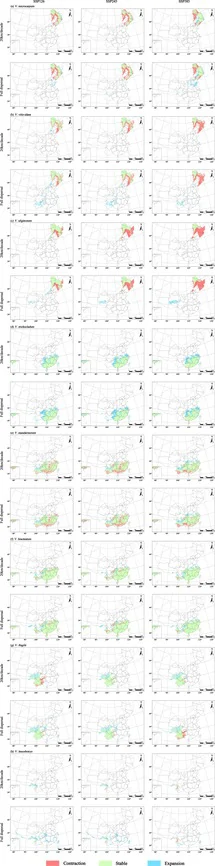
**FIGURE 4.** The changes in suitable areas of eight *Vaccinium* berry wild relatives in China between future and current potential distributions under three emission scenarios (SSP126, SSP245 and SSP585) and two dispersal scenarios (20 km/decade and full dispersal).

We apologize for this error.

