# Supplementary material for: Distribution Shifts of Vaccinium Berry Wild Relatives in China Under Climate and Land‐Use Changes
Source: Ecol Evol. 2026 Aug 9;16(8):e74154. doi: 10.1002/ece3.74154 (PMC13453431; doi:10.1002/ece3.74154)
Supplement: Supplementary file 1 [file ECE3-16-e74154-s001.docx]

**Appendix. Supporting information**

**Table S1** Species selection criteria and supporting references.

| Species | Selection criterion | Supporting reference(s) | |
| --- | --- | --- | --- |
| *V*. *microcarpum* | Edible value | Tundis, R., Tenuta, M. C., Loizzo, M. R., Bonesi, M., Finetti, F., Trabalzini, L. & Deguin, B. (2021) *Vaccinium* Species (Ericaceae): From Chemical Composition to Bio-Functional Activities. Applied Sciences, 11, 5655. | |
| *V*. *vitis-idaea* | Edible value, Economic importance | Fang, R. Z., Xu, T. Z., Huang, S. H., et al. (1991) Flora Reipublicae Popularis Sinicae, 57(3): 108–200. | |
| *V*. *uliginosum* | Edible value, Economic importance | Fang, R. Z., Xu, T. Z., Huang, S. H., et al. (1991) Flora Reipublicae Popularis Sinicae, 57(3): 108–200;  Wang, Y. S. (2016) Diversity of wild *Vaccinium* resources in China and recent research advances. Northern Horticulture, (04), 191–197. | |
| *V*. *trichocladum* | Ornamental and medicinal value | Huang, H. Q. (2013) Investigation of wild Ericaceae plant resources in Youxi Jiufu Mountain Provincial Nature Reserve. Jiangxi; Forestry Science and Technology, (4), 20–23; China National Corporation of Traditional & Herbal Medicine. (1994) A Compendium of Chinese Medicinal Material Resources. Beijing: Science Press. | |
| *V*. *mandarinorum* | Documented wild resources and folk utilization in Fujian | Hu, H. Y. & Ma, Z. J. (2001) Wild *Vaccinium* resources and their folk utilization in Fujian Province. Subtropical Plant Science, (1), 49–53. | |
| *V*. *bracteatum* | Edible and medicinal value, Economic importance | Wang, Y. S. (2016) Diversity of wild *Vaccinium* resources in China and recent research advances. Northern Horticulture, (04), 191–197. |  |
| *V*. *fragile* | Edible and medicinal value | Kunming Institute of Botany, Chinese Academy of Sciences. (1991) Flora of Yunnan, Vol. 5. Beijing: Science Press. |  |
| *V*. *leucobotrys* | Edible and ornamental value | Bi, H. L., Yang, Z. S., Yang, H. T., He, G. Q., Yang, Y. L. & He, Z. J. (2018) Tissue culture and rapid propagation of *Vaccinium leucobotrys*. Acta Agriculturae Jiangxi, 30(5), 23–27. |  |

**Table S2** Occurrence number for eight *Vaccinium* berry wild relatives.

| Species | Occurrence number |
| --- | --- |
| *V*. *microcarpum* | 29 |
| *V*. *vitis-idaea* | 71 |
| *V*. *uliginosum* | 78 |
| *V*. *trichocladum* | 49 |
| *V*. *mandarinorum* | 299 |
| *V*. *bracteatum* | 542 |
| *V*. *fragile* | 119 |
| *V*. *leucobotrys* | 26 |
| Total | 1213 |

**Table S3** Pearson correlation coefficient matrix between selected climate variables.

|  | Bio5 | Bio15 | Bio18 | Bio19 |
| --- | --- | --- | --- | --- |
| Bio1 | 0.65 | -0.62 | 0.41 | 0.50 |
| Bio5 |  | -0.39 | -0.08 | 0.28 |
| Bio15 |  |  | -0.18 | -0.76 |
| Bio18 |  |  |  | 0.48 |

Bio1: Annual mean temperature; Bio5: Max temperature of warmest month; Bio15: Precipitation seasonality; Bio18: Precipitation of warmest quarter; Bio19: Precipitation of coldest quarter.

**Table S4** The performance of species distribution models for eight *Vaccinium* berry wild relatives. True skills statistics (the average of the five modeling approaches) are used in the current summary. Higher values indicate a higher predictive power of the models.

| Species | TSS values |
| --- | --- |
| *V*. *microcarpum* | 0.889 |
| *V*. *vitis-idaea* | 0.841 |
| *V*. *uliginosum* | 0.885 |
| *V*. *trichocladum* | 0.936 |
| *V*. *mandarinorum* | 0.837 |
| *V*. *bracteatum* | 0.799 |
| *V*. *fragile* | 0.918 |
| *V*. *leucobotrys* | 0.955 |

**Table S5** The range size of the potential current suitable areas for eight *Vaccinium* berry wild relatives.

| Species | Range size (km²) | | | |
| --- | --- | --- | --- | --- |
|  | Less suitable | Moderately suitable | Highly suitable | Total |
| *V. microcarpum* | 3.43×10^5^ | 1.94×10^5^ | 2.99×10^5^ | 8.36×10^5^ |
| *V. vitis-idaea* | 1.63×10^5^ | 1.99×10^5^ | 4.51×10^5^ | 8.13×10^5^ |
| *V. uliginosum* | 1.68×10^5^ | 2.09×10^5^ | 5.83×10^5^ | 9.61×10^5^ |
| *V. trichocladum* | 2.39×10^5^ | 3.68×10^5^ | 3.61×10^5^ | 9.68×10^5^ |
| *V. mandarinorum* | 4.19×10^5^ | 5.35×10^5^ | 5.73×10^5^ | 1.53×10^6^ |
| *V. bracteatum* | 4.29×10^5^ | 6.34×10^5^ | 6.80×10^5^ | 1.74×10^6^ |
| *V. fragile* | 1.72×10^5^ | 2.38×10^5^ | 3.72×10^5^ | 7.81×10^5^ |
| *V. leucobotrys* | 2.10×10^4^ | 2.65×10^4^ | 4.92×10^4^ | 9.67×10^4^ |

**Table S6** Mean relative importance of each variable to the final SDMs of eight *Vaccinium* berry wild relatives. The values highlighted in bold are greater than 20% were selected as the main environmental factors for each species.

| Environmental variables | *V. microcarpum* | *V. vitis-idaea* | *V. uliginosum* | *V. trichocladum* |
| --- | --- | --- | --- | --- |
| Bio1 | 0.192±0.037 | 0.182±0.012 | **0.342±0.021** | **0.383±0.028** |
| Bio5 | 0.098±0.022 | 0.134±0.018 | 0.096±0.012 | 0.032±0.009 |
| Bio15 | **0.353±0.046** | **0.348±0.021** | 0.164±0.017 | 0.066±0.016 |
| Bio18 | 0.089±0.024 | 0.041±0.002 | **0.209±0.016** | **0.204±0.025** |
| Bio19 | 0.076±0.023 | 0.103±0.013 | 0.026±0.005 | 0.192±0.025 |
| Elevation | 0.092±0.025 | 0.111±0.014 | 0.066±0.009 | 0.046±0.010 |
| Barren | 0.014±0.009 | 0.059±0.004 | 0.100±0.016 | 0.026±0.013 |
| Cropland | 0.018±0.005 | 0.079±0.016 | 0.037±0.012 | 0.048±0.012 |
| Forest | **0.310±0.043** | 0.117±0.020 | 0.092±0.016 | 0.055±0.014 |
| Grass | **0.314±0.050** | 0.050±0.011 | 0.033±0.010 | 0.035±0.011 |
| UrbanGreenSpace | 0.003±0.024 | 0.023±0.022 | 0.158±0.017 | 0.136±0.019 |
| Impervious | 0.127±0.039 | 0.072±0.018 | 0.087±0.018 | 0.046±0.014 |
| Shrub | 0.056±0.019 | 0.060±0.016 | 0.047±0.012 | 0.060±0.019 |
| Wetland | 0.000 | 0.005±0.010 | 0.064±0.001 | 0.083±0.015 |

| Environmental variables | *V. mandarinorum* | *V. bracteatum* | *V. fragile* | *V. leucobotrys* |
| --- | --- | --- | --- | --- |
| Bio1 | **0.246±0.018** | 0.134±0.011 | **0.205±0.019** | 0.006±0.001 |
| Bio5 | 0.047±0.006 | 0.018±0.003 | 0.107±0.013 | 0.131±0.028 |
| Bio15 | 0.042±0.005 | 0.029±0.003 | 0.097±0.013 | 0.009±0.001 |
| Bio18 | **0.288±0.024** | 0.072±0.007 | 0.043±0.002 | **0.389±0.044** |
| Bio19 | 0.128±0.013 | **0.337±0.224** | 0.163±0.014 | **0.219±0.046** |
| Elevation | 0.040±0.004 | 0.021±0.003 | 0.127±0.012 | **0.328±0.045** |
| Barren | 0.052±0.010 | 0.105±0.011 | 0.030±0.007 | 0.003±0.001 |
| Cropland | 0.067±0.009 | 0.036±0.004 | 0.077±0.011 | 0.023±0.011 |
| Forest | 0.087±0.013 | 0.022±0.003 | 0.164±0.017 | **0.299±0.045** |
| Grass | 0.028±0.005 | 0.031±0.003 | 0.085±0.010 | 0.021±0.013 |
| UrbanGreenSpace | 0.034±0.006 | 0.019±0.003 | 0.072±0.011 | 0.000 |
| Impervious | 0.038±0.006 | 0.039±0.003 | 0.040±0.008 | 0.000 |
| Shrub | 0.031±0.005 | 0.001±0.002 | 0.039±0.006 | 0.020±0.013 |
| Wetland | 0.022±0.004 | 0.022±0.005 | 0.018±0.008 | 0.000 |

**Table S7** The range change radio for eight *Vaccinium* berry wild relatives.

| Specie | Climate scenario | Dispersal scenario | Range change radio (%) |
| --- | --- | --- | --- |
| *V. microcarpum* | SSP12670 | 20km/decade | -34.817 |
| *V. microcarpum* | SSP24570 | 20km/decade | -45.241 |
| *V. microcarpum* | SSP58570 | 20km/decade | -22.936 |
| *V. microcarpum* | SSP12670 | full | -27.295 |
| *V. microcarpum* | SSP24570 | full | -41.259 |
| *V. microcarpum* | SSP58570 | full | -4.873 |
| *V. vitis-idaea* | SSP12670 | 20km/decade | -24.769 |
| *V. vitis-idaea* | SSP24570 | 20km/decade | -37.197 |
| *V. vitis-idaea* | SSP58570 | 20km/decade | -61.609 |
| *V. vitis-idaea* | SSP12670 | full | 5.623 |
| *V. vitis-idaea* | SSP24570 | full | -12.858 |
| *V. vitis-idaea* | SSP58570 | full | -43.734 |
| *V. uliginosum* | SSP12670 | 20km/decade | -26.067 |
| *V. uliginosum* | SSP24570 | 20km/decade | -50.125 |
| *V. uliginosum* | SSP58570 | 20km/decade | -70.55 |
| *V. uliginosum* | SSP12670 | full | -17.198 |
| *V. uliginosum* | SSP24570 | full | -40.256 |
| *V. uliginosum* | SSP58570 | full | -53.206 |
| *V. trichocladum* | SSP12670 | 20km/decade | 33.058 |
| *V. trichocladum* | SSP24570 | 20km/decade | 39.585 |
| *V. trichocladum* | SSP58570 | 20km/decade | 35.815 |
| *V. trichocladum* | SSP12670 | full | 45.378 |
| *V. trichocladum* | SSP24570 | full | 55.985 |
| *V. trichocladum* | SSP58570 | full | 37.457 |
| *V. mandarinorum* | SSP12670 | 20km/decade | -14.542 |
| *V. mandarinorum* | SSP24570 | 20km/decade | -17.935 |
| *V. mandarinorum* | SSP58570 | 20km/decade | -4.422 |
| *V. mandarinorum* | SSP12670 | full | -13.867 |
| *V. mandarinorum* | SSP24570 | full | -17.228 |
| *V. mandarinorum* | SSP58570 | full | -3.871 |
| *V. bracteatum* | SSP12670 | 20km/decade | 1.63 |
| *V. bracteatum* | SSP24570 | 20km/decade | -1.515 |
| *V. bracteatum* | SSP58570 | 20km/decade | 7.179 |
| *V. bracteatum* | SSP12670 | full | 2.766 |
| *V. bracteatum* | SSP24570 | full | -0.436 |
| *V. bracteatum* | SSP58570 | full | 7.724 |
| *V. fragile* | SSP12670 | 20km/decade | 19.724 |
| *V. fragile* | SSP24570 | 20km/decade | 21.195 |
| *V. fragile* | SSP58570 | 20km/decade | -5.388 |
| *V. fragile* | SSP12670 | full | 30.360 |
| *V. fragile* | SSP24570 | full | 30.398 |
| *V. fragile* | SSP58570 | full | -2.189 |
| *V. leucobotrys* | SSP12670 | 20km/decade | 64.012 |
| *V. leucobotrys* | SSP24570 | 20km/decade | 40.228 |
| *V. leucobotrys* | SSP58570 | 20km/decade | -11.996 |
| *V. leucobotrys* | SSP12670 | full | 322.234 |
| *V. leucobotrys* | SSP24570 | full | 292.244 |
| *V. leucobotrys* | SSP58570 | full | 58.221 |

**Table S8** Centroid shifts in direction and distance of eight *Vaccinium* berry wild relatives between current and projected distributions under climate and land use changes scenarios with two dispersal scenarios.

| Species | Climate scenario | Dispersal scenario | Distance (km) | Degrees (°) |
| --- | --- | --- | --- | --- |
| *V. microcarpum* | SSP126 | 20km/decade | 58 | 81.61 |
| *V. microcarpum* | SSP245 | 20km/decade | 162 | 78.90 |
| *V. microcarpum* | SSP585 | 20km/decade | 52 | 140.39 |
| *V. microcarpum* | SSP126 | full | 53 | 130.79 |
| *V. microcarpum* | SSP245 | full | 253 | 113.29 |
| *V. microcarpum* | SSP585 | full | 39 | 176.44 |
| *V. vitis-idaea* | SSP126 | 20km/decade | 106 | 341.26 |
| *V. vitis-idaea* | SSP245 | 20km/decade | 335 | 339.44 |
| *V. vitis-idaea* | SSP585 | 20km/decade | 153 | 335.27 |
| *V. vitis-idaea* | SSP126 | full | 123 | 258.12 |
| *V. vitis-idaea* | SSP245 | full | 283 | 275.87 |
| *V. vitis-idaea* | SSP585 | full | 139 | 299.36 |
| *V. uliginosum* | SSP126 | 20km/decade | 109 | 331.24 |
| *V. uliginosum* | SSP245 | 20km/decade | 408 | 336.43 |
| *V. uliginosum* | SSP585 | 20km/decade | 244 | 337.21 |
| *V. uliginosum* | SSP126 | full | 121 | 295.07 |
| *V. uliginosum* | SSP245 | full | 599 | 300.23 |
| *V. uliginosum* | SSP585 | full | 253 | 270.77 |
| *V. trichocladum* | SSP126 | 20km/decade | 65 | 349.50 |
| *V. trichocladum* | SSP245 | 20km/decade | 115 | 352.91 |
| *V. trichocladum* | SSP585 | 20km/decade | 81 | 15.66 |
| *V. trichocladum* | SSP126 | full | 98 | 322.96 |
| *V. trichocladum* | SSP245 | full | 117 | 324.58 |
| *V. trichocladum* | SSP585 | full | 114 | 15.19 |
| *V. mandarinorum* | SSP126 | 20km/decade | 57 | 316.00 |
| *V. mandarinorum* | SSP245 | 20km/decade | 90 | 358.95 |
| *V. mandarinorum*  *V. mandarinorum* | SSP585  SSP126 | 20km/decade  full | 67  58 | 353.89  314.67 |
| *V. mandarinorum* | SSP245 | full | 90 | 356.50 |
| *V. mandarinorum* | SSP585 | full | 68 | 353.21 |
| *V. bracteatum* | SSP126 | 20km/decade | 46 | 279.06 |
| *V. bracteatum* | SSP245 | 20km/decade | 39 | 335.23 |
| *V. bracteatum* | SSP585 | 20km/decade | 12 | 336.58 |
| *V. bracteatum* | SSP126 | full | 65 | 277.49 |
| *V. bracteatum* | SSP245 | full | 42 | 295.98 |
| *V. bracteatum* | SSP585 | full | 27 | 330.11 |
| *V. fragile* | SSP126 | 20km/decade | 96 | 315.92 |
| *V. fragile* | SSP245 | 20km/decade | 183 | 327.62 |
| *V. fragile* | SSP585 | 20km/decade | 112 | 315.79 |
| *V. fragile* | SSP126 | full | 73 | 341.92 |
| *V. fragile* | SSP245 | full | 167 | 347.55 |
| *V. fragile* | SSP585 | full | 100 | 319.57 |
| *V. leucobotrys* | SSP126 | 20km/decade | 57 | 354.71 |
| *V. leucobotrys* | SSP245 | 20km/decade | 73 | 2.63 |
| *V. leucobotrys* | SSP585 | 20km/decade | 54 | 268.28 |
| *V. leucobotrys* | SSP126 | full | 439 | 78.83 |
| *V. leucobotrys* | SSP245 | full | 664 | 78.86 |
| *V. leucobotrys* | SSP585 | full | 495 | 97.41 |

**Table S9** Persistent suitable areas for eight *Vaccinium* berry wild relatives.

| Species | Persistent suitable areas (km²) |
| --- | --- |
| *V*. *microcarpum* | 3.35×10^5^ |
| *V*. *vitis-idaea* | 3.04×10^5^ |
| *V*. *uliginosum* | 1.97×10^5^ |
| *V*. *trichocladum* | 9.43×10^5^ |
| *V*. *mandarinorum* | 8.55×10^5^ |
| *V*. *bracteatum* | 1.42×10^6^ |
| *V*. *fragile* | 5.86×10^5^ |
| *V*. *leucobotrys* | 4.25×10^4^ |
